# Phase 1 trial of tivantinib in combination with sorafenib in adult patients with advanced solid tumors

**DOI:** 10.1007/s10637-014-0167-5

**Published:** 2014-10-08

**Authors:** Igor Puzanov, Jeffrey Sosman, Armando Santoro, Muhammad W. Saif, Laura Goff, Grace K. Dy, Paolo Zucali, Julie A. Means-Powell, Wen Wee Ma, Matteo Simonelli, Robert Martell, Feng Chai, Maria Lamar, Ronald E. Savage, Brian Schwartz, Alex A. Adjei

**Affiliations:** 1 Vanderbilt University Medical Center, 2220 Pierce Avenue, 777 Preston Research Building, Nashville, TN USA; 2 Humanitas Cancer Center IRCCS, Rozzano, MI Italy; 3 Tufts Medical Center, Boston, MA USA; 4 Roswell Park Cancer Institute, Buffalo, NY USA; 5 ArQule, Inc., Woburn, MA USA

**Keywords:** Tivantinib, Sorafenib, Phase I trial, VEGF inhibition, MET RTK inhibition, Advanced tumors

## Abstract

*Purpose* This phase I study evaluated the safety, tolerability, maximum tolerated dose (MTD), and recommended phase II dose (RP2D) of tivantinib combined with sorafenib in patients with advanced solid tumors. *Materials and Methods* A standard 3 + 3 dose escalation design was used. At the RP2D, expansion cohorts in 5 tumor types could be enrolled. Pharmacogenetic and pharmacodynamic analysis were performed. *Results* Eighty-seven patients received the study treatment. The combination had no unexpected toxicities. The most common treatment-related adverse events (AE) were rash (40 %), diarrhea (38 %), and anorexia (33 %). The RP2D was tivantinib 360 mg BID and sorafenib 400 mg BID for all cancer histologies, except in hepatocellular carcinoma (HCC) patients tivantinib was 240 mg BID plus sorafenib 400 mg BID. The overall response rate was 12 % in all patients, 26 % in melanoma, 15 % in renal cell carcinoma (RCC), 10 % in HCC, and 0 % in other patients. Disease control rate (CR, PR and SD ≥8 weeks) was 58 % in all patients, 90 % in RCC, 65 % in HCC, 63 % in melanoma, 40 % in breast cancer, and 8 % in NSCLC patients. *Conclusions* The combination treatment could be administered at full standard single-agent doses in all patients except those with HCC, where tivantinib was lowered to 240 mg BID. Preliminary evidence of anticancer activity was observed in patients with RCC, HCC, and melanoma, including patients refractory to sorafenib and/or other anti-VEGF pathway therapies. The combination treatment has therapeutic potential in treating a variety of solid tumors.

## Introduction

Dysregulation of MET receptor tyrosine kinase (RTK), the only known high-affinity receptor for hepatocyte growth factor (HGF), has been implicated in tumor cell proliferation, migration, invasiveness, angiogenesis, and metastasis in a broad spectrum of human cancers [1, 2]. MET pathway is one of the most dysregulated pathways in human cancer with MET over-expression, amplification and/or mutations [3, 4, 2, 1]. MET signaling results in activation of downstream pathways, including focal adhesion kinase, Ras/Raf/MEK/ERK, and phosphatidylinositol-3-kinase/AKT pathways. High-level MET amplification is an established mechanism of resistance to EGFR tyrosine kinase inhibitors (TKIs) among patients with EGFR-mutant NSCLC [5].

Tivantinib (ARQ 197; ArQule, Inc., Woburn, MA, US; Daiichi Sankyo Co., Ltd., Tokyo, Japan) is a selective, oral, small molecule inhibitor of the MET RTK [6]. Tivantinib has demonstrated antitumor activity in a wide range of human tumor cell lines and in xenograft models of human cancers [7]. Studies have identified MET as a mediator of tumor invasion and resistance following angiogenesis inhibition [8, 9]. Combination of tivantinib and sorafenib has resulted in additive or synergistic inhibition of cell proliferation in several human tumor cell lines [10]. Phase 1 and 2 single agent and combination studies of tivantinib have demonstrated a manageable safety profile and preliminary antitumor activity in a range of cancer types [11–16]. Up to 87 % of RCC tumors over-express HGF/MET [17–19] 20–48 % of HCC tumors over-express MET, which is associated with a significantly shorter survival rate [20, 21]. MET is also expressed in 88 % and activated in 21 % of melanoma tumors [22]. NRAS-mutated melanoma cell lines are sensitive to MET inhibition with regards to proliferation, migration and apoptosis [23].

This Phase 1 study (ARQ 197–116) was initiated based on the importance and prevalence of MET expression/over-expression in many cancer types and evidence of synergistic inhibition of tivantinib and sorafenib in vivo in several cancer cell lines [10].

## Materials and methods

### Study design and dosing

This study was conducted at 3 centers in the U.S. and 1 center in Italy.

Dose-escalation followed the traditional 3 + 3 scheme. Two dose levels were evaluated in 28-day cycles: level 1 (tivantinib 360 mg BID/sorafenib 200 mg BID) and level 2 (tivantinib 360 mg BID/sorafenib 400 mg BID). Tivantinib dose of 360 mg BID was selected based on earlier phase I studies that had established this dose as the RP2D [12]. Intra-patient dose escalation to the RP2D was allowed. After RP2D was determined, expansion cohorts with selected tumor types were enrolled and treated, including up to 20 patients each with unresectable HCC, advanced RCC or melanoma (at least 10 of whom had NRAS mutation); and up to ten patients each with breast cancer or NSCLC. Treatment continued until unacceptable toxicity, progressive disease (PD) or another discontinuation criterion was met. The tivantinib starting dose was reduced to 240 mg BID for patients with HCC after a safety review of HCC patients in a concurrent phase II study of single agent tivantinib revealed an increase in the incidence of severe (≥Grade 3) neutropenia [14] and a report of febrile neutropenia. If a patient with HCC tolerated the 240 mg starting dose for at least one cycle, the dose was allowed to be increased to 360 mg BID.

### Safety

Drug safety was evaluated at baseline, weekly during the 1^st^ cycle, then once per cycle until 30 days after last dose of study treatment. All AEs and laboratory variables were assessed using Common Terminology Criteria for Adverse Events (CTCAE) (version 3.0) [24].

### Definition of DLT and MTD

DLT was defined as any of the following toxicities, related to tivantinib and/or sorafenib within the first cycle : Grade 4 absolute neutrophil count (ANC) or Grade 3 thrombocytopenia in the presence of bleeding; Grade 3 or worse non-hematological toxicity of any duration except alopecia (any grade); Grade 3 or 4 nausea/vomiting or diarrhea despite optimal medical management and reversible laboratory abnormalities with no clinical sequelae and/or no clinical significance in the opinion of an Investigator; and any other toxicity that in the view of the Principal Investigator represented a clinically significant hazard to the patient. MTD was defined as the highest dose at which <33 % of six patients experienced a DLT.

### Patient selection

Patients were enrolled if they met the following criteria: were 18 years of age or older; had advanced or metastatic solid tumors for which no effective treatment was available; signed informed consent; had Eastern Cooperative Oncology group (ECOG) performance status (PS) ≤1 [19]; had adequate bone marrow, liver and renal functions; received no previous anti-cancer therapy within 4 weeks prior to the first day of study drug; had no history of congestive heart failure defined as Class II to IV per New York Heart Association classification or active coronary heart disease; had no previously diagnosed clinically significant bradycardia, other uncontrolled cardiac arrhythmia defined as ≥ Grade 2 according to CTCAE v. 4.0. 3.0 [24], uncontrolled hypertension or myocardial infarction within 6 months of first dose of study drugs; had no other significant comorbidities. Patients who were on sorafenib monotherapy treatment could be enrolled at the discretion of the investigators.

### Pharmacogenetics and pharmacodynamic studies

One blood sample was collected on Cycle 1 Day 1 for detecting CYP2C19 polymorphism (CYP450 AmpliChip; Roche, Basel, Switzerland) and the analyses were conducted at Solstas Lab Partners, Greensboro, NC.

Archival tumor tissue samples were collected at baseline if available. Immunohistochemical (IHC) tests of total MET expression was performed by the sponsor and confirmed by a CLIA-certified central laboratory using the Ventana CONFIRM anti-total MET (SP44) rabbit monoclonal antibody (Ventana Medical Systems, Inc., Tucson, AZ). Sponsor’s tests were conducted on small batches on an ongoing basis. CLIA-certified central laboratory tests were performed as one batch at the end of patient enrollment. When a discrepancy occurred, to avoid effects caused by MET degeneration over time, the sponsor′s results were used if the tissue sections tested by the central lab were prepared at the site and stored as slides. Slides were scanned using a digital imaging system (Aperio Technologies, Vista, CA), and a board certified pathologist scored images. Staining intensity was scored on a scale of 0, 1+, 2+, 3+. Samples that scored ≥ 2+ in ≥ 50 % of tumor cells were defined MET-high. Others were defined MET-low.

### Pharmacokinetic (PK) Analysis

Blood samples were collected pre-, 1, 2, 4, 6, 8, 12, and 24 h post-dose on Days 1 and 2 of Cycles 1 and 2. The day before and on the day of PK sampling, tivantinib and sorafenib were administered at the same time. Noncompartmental PK parameters were calculated using WinNonlin (Pharsight, Mountain View, CA).

### Tumor response

Tumor measurements were performed at baseline and in 8-week intervals. Tumor responses were evaluated based on RECIST 1.1 by the investigators.

### Statistical analysis

Continuous measurements were summarized by mean (±SD) or median. Categorical data were summarized by frequency and percentages. Progression-free survival (PFS) analysis used the Kaplan-Meier method. PFS was calculated from the first dose until PD according to RECIST 1.1 or death.

## Results

### Patient populations

Eighty-seven patients were enrolled between June 2009 and March 2012 (see Table 1 for demographics and baseline characteristics). Fourteen of 20 (70.0 %) RCC patients received a prior anti-VEGF treatment [16 (80.0 %) were clear cell carcinoma, three (15.0 %) papillary and one (5.0 %) clear cell + chromophobe RCC]. Eight of 20 (40.0 %) patients with HCC had a prior anti-VEGF treatment and 14 (70.0 %) of them were Child-Pugh A. Ten of 19 (52.6 %) melanoma patients had NRAS mutation, one (5.3 %) was wild type, and eight (42.1 %) were unknown.

**Table 1:** Patient demographics and clinical characteristics

Characteristic	Cancer Type						
	RCC (*N* = 20)	HCC (*N* = 20)	Melanoma (*N* = 19)	NSCLC (*N* = 12)	Breast (*N* = 10)	Other* (*N* = 6)	All patients (*N* = 87)
Age (years)							
Mean (SD)	59.5 (12.9)	61.9 (9.1)	65.1 (12.5)	59.8 (6.5)	54.5 (10.8)	59.7 (13.8)	60.7 (11.2)
Min, Max	23, 75	41, 77	31, 85	48, 69	34, 68	41, 77	23, 85
Gender, *n* (%)							
Female	4 (20.0)	4 (20.0)	2 (10.5)	3 (25.0)	10 (100.0)	1 (16.7)	24 (27.6)
Male	16 (80.0)	16 (80.0)	17 (89.5)	9 (75.0)	0	5 (83.3)	63 (72.4)
Race, *n* (%)							
White	19 (95.0)	17 (85.0)	18 (94.7)	12 (100.0)	10 (100.0)	6 (100.0)	82 (94.3)
Black or African American	1 (5.0)	1 (5.0)	0	0	0	0	2 (2.3)
Asian	0	2 (10.0)	0	0	0	0	2 (2.3)
Other	0	0	1 (5.3)	0	0	0	1 (1.1)
Baseline ECOG Performance Status, *n* (%)							
0	14 (70.0)	13 (65.0)	7 (36.8)	5 (41.72)	4 (40.0)	1 (16.7)	44 (50.6)
1	6 (30.0)	7 (35.0)	12 (63.2)	7 (58.3)	6 (60.0)	5 (83.3)	43 (49.4)
Tumor Stage at study entry, n (%)							
Stage IIB	0	1 (5.0)	0	0	0	0	1 (1.1)
Stage IIIA	0	2 10.0)	0	0	0	0	2 (2.3)
Stage IIIB	1 (5.0)	2 (10.0)	0	0	0	0	3 (3.4)
Stage IV	19 (95.0)	15 (75.0)	19 (100.0)	12 (100.0)	10 (100.0)	6 (100.0)	81 (93.1)
Number of prior anti-cancer systemic regimens							
Median	2.0	1.0	2.0	3.5	6.0	5.0	2.0
Min, Max	0, 4	0, 5	0, 6	1, 7	4, 13	2, 7	0, 13
Prior anti-VEGF Therapy, *n* (%)							
Yes (at least one)	14 (70.0)	8 (40.0)					
No	6 (30.0)	12 (80.0)					
Liver Child-Pugh Status, *n* (%)							
Child-Pugh A		14 (70.0)					
Child-Pugh B		6 (30.0)					
RCC subtype, *n* (%)							
Clear cell	16 (80.0)						
Papillary	3 (15.0)						
Clear cell/chromophobe	1 (5.0)						
Tumor *NRAS* subtype, *n* (%)							
Mutant			10 (52.6)				
Wild type			1 (5.3)				
Unknown			8 (42.1)				

Abbreviations: *ECOG PS* Eastern Cooperative Group performance status

*adenocarcinoma of rectum (1), adenocarcinoma of colon (2), adenocarcinoma of esophagus (1), mesothelioma (2)

### Treatment duration

The median durations of exposure for tivantinib in all patients, RCC, HCC, melanoma, NSCLC, and breast cancer patients were 108 (range 1–822), 225 (51–822), 93 (20–483), 112 (7–391), 69 (1–112), and 53 (14–233) days, respectively. The median durations of exposure for sorafenib in all patients, RCC, HCC, melanoma, NSCLC, and breast cancer patients were 104 (range 1–822), 225 (51–822), 87 (20–483), 112 (7–391), 60 (1–112) and 45 (8–233) days, respectively.

### DLTs and RP2D

No DLT was observed among the five patients treated at dose level 1 (tivantinib 360 mg BID/sorafenib 200 mg BID). One of the first six patients (16.7 %) treated at dose level 2 (tivantinib 360 mg BID/sorafenib 400 mg BID) experienced a DLT of grade 3 atrial fibrillation. Dose level 2 was determined as RP2D. Seventy-six additional patients in expansion cohorts were treated at RP2D. Twelve of the 76 patients (15.8 %) experienced 18 serious AEs that met criteria defined as DLTs, including four grade 3 palmar-plantar erythrodysaesthesia syndrome, three grade 3 rash, and one each of grade 3 dyspnea, fatigue, hypertension, pain in extremity, dizziness, prolonged prothrombin time/increased international normalized ratio, neutropenia, diarrhea, pneumonia and allodynia.

Following the increased rates of myelo-toxicities reported in patients with HCC treated with tivantinib monotherapy in another study [14], plus an event of grade 3 febrile neutropenia in a HCC patient in this study treated at an initial dose of 360 mg tivantinib plus 400 mg sorafenib, the tivantinib starting dose was reduced to 240 mg BID for HCC patients with the option to increase to 360 mg BID based on tolerability, resulting in 10 patients treated at initial dose of 360 mg BID and 10 treated at 240 mg BID with tivantinib. Thus, the RP2D for HCC patients was determined as tivantinib 240 mg BID and sorafenib 400 mg BID.

### Safety and tolerability

Table 2 displays the treatment-related AEs related to tivantinib and/or sorafenib that occurred in at least 5 % of all patients. The most common AEs included rash (40.2 %), diarrhea (37.9 %), anorexia (33.3 %), fatigue (31.0 %), alopecia (25.3 %), palmar-plantar erythrodysaesthesia syndrome (21.8 %), and weight reduction (20.7 %). Overall, the treatment-related toxicities were mainly grade 1 or 2.

**Table 2:** Treatment Related Adverse Events in ≥5 % of Patients

System organ class preferred term, *n* (%)	RCC (*N* = 20)	HCC (*N* = 20)	Melanoma (*N* = 19)	NSCLC (*N* = 12)	Breast (*N* = 10)	Other (*N* = 6)	All Patients (*N* = 87)
Number of Patients with at least one Drug Related AE	20 (100.0)	20 (100.0)	18 (94.7)	9 (75.0)	9 (90.0)	5 (83.3)	81 (93.1)
Blood and lymphatic system disorders							
Anemia		4 (20.0)	2 (10.5)				6 (6.9)
Leukopenia	1 (5.0)	1 (5.0)	4 (21.1)		1 (10.0)		7 (8.0)
Lymphopenia	2 (10.0)	1 (5.0)	3 (15.8)		1 (10.0)	2 (33.3)	9 (10.3)
Neutropenia		2 (10.0)	2 (10.5)				4 (4.6)
Thrombocytopenia	1 (5.0)	1 (5.0)	2 (10.5)		2 (20.0)		6 (6.9)
Gastrointestinal disorders							
Diarrhea	10 (50.0)	8 (40.0)	9 (47.4)	1 (8.3)	4 (40.0)	1 (16.7)	33 (37.9)
Nausea	2 (10.0)	5 (25.0)	7 (36.8)	2 (16.7)			16 (18.4)
Stomatitis	6 (30.0)	2 (10.0)	4 (21.1)		2 (20.0)		14 (16.1)
Vomiting	1 (5.0)	3 (15.0)	3 (15.8)	2 (16.7)	1 (10.0)		10 (11.5)
General disorders and administration site conditions							
Fatigue	5 (25.0)	7 (35.0)	8 (42.1)	2 (16.7)	1 (10.0)	4 (66.7)	27 (31.0)
Asthenia	4 (20.0)	1 (5.0)					5 (5.7)
Mucosal inflammation		2 (10.0)	3 (15.8)				5 (5.7)
Investigations							
Alanine aminotransferase increased	1 (5.0)	1 (5.0)	2 (10.5)		1 (10.0)		5 (5.7)
Aspartate aminotransferase increased	1 (5.0)	1 (5.0)	2 (10.5)		2 (20.0)		6 (6.9)
Hemoglobin decreased	2 (10.0)	1 (5.0)	3 (15.8)				6 (6.9)
Weight decreased	4 (20.0)	4 (20.0)	6 (31.6)	2 (16.7)	1 (10.0)	1 (16.7)	18 (20.7)
Metabolism and nutrition disorders							
Anorexia	5 (25.0)	7 (35.0)	7 (36.8)	5 (41.7)	2 (20.0)	3 (50.0)	29 (33.3)
Hyperuricemia	2 (10.0)	1 (5.0)	2 (10.5)				5 (5.7)
Hypoalbuminemia	2 (10.0)		3 (15.8)				5 (5.7)
Hypophosphatemia	8 (40.0)	1 (5.0)	4 (21.1)	1 (8.3)	1 (10.0)	0	15 (17.2)
Skin and subcutaneous tissue disorders							
Alopecia	9 (45.0)	6 (30.0)	6 (31.6)		1 (10.0)		22 (25.3)
Dermatitis acneiform	5 (25.0)	2 (10.0)	2 (10.5)	1 (8.3)	1 (10.0)		11 (12.6)
Dry skin	2 (10.0)	1 (5.0)	1 (5.3)	2 (16.7)	2 (20.0)		8 (9.2)
Pain of skin	4 (20.0)	1 (5.0)	1 (5.3)				6 (6.9)
Palmar-plantar erythrodysaesthesia syndrome	5 (25.0)	8 (40.0)	1 (5.3)	2 (16.7)	2 (20.0)	1 (16.7)	19 (21.8)
Pruritus	5 (25.0)	1 (5.0)	3 (15.8)		1 (10.0)		10 (11.5)
Rash	13 (65.0)	7 (35.0)	10 (52.6)	3 (25.0)	2 (20.0)		35 (40.2)
Vascular disorders							
Flushing	2 (10.0)		3 (15.8)		1 (10.0)		6 (6.9)
Hypertension	3 (15.0)	2 (10.0)	5 (26.3)	1 (8.3)	3 (30.0)		14 (16.1)

One of 10 HCC patients treated at initial tivantinib dose of 360 mg BID experienced grade 3 neutropenia that led to dose reduction, and another experienced grade 3 febrile neutropenia that led to treatment discontinuation. One of 10 HCC patients treated at initial tivantinib dose of 240 mg BID developed grade 3 neutropenia that led to tivantinib dose reduction to 120 mg BID. This patient later experienced additional episodes of grade 4 neutropenia and neutropenic infection. None of the remaining nine patients at 240 mg BID dose had experienced neutropenia, including two who had tivantinib dose increased to 360 mg BID after one cycle.

### Pharmacokinetic analyses

PK data are summarized in Table 3. In non-HCC patients receiving tivantinib at 360 mg BID and sorafenib at 400 mg BID, the mean C_max_ and AUC_0-12h_ of tivantinib in steady state (cycle 2) were 1,323 ± 1,043 ng/mL (*n* = 33) and 8,737 ± 10,759 ng*hr/mL (*n* = 22), respectively. For sorafenib, they were 6,757 ± 3,725 ng/mL (*n* = 33) and 39,906 ± 20,604 ng*hr/mL (*n* = 14), respectively.

**Table 3:** Steady State PK Parameters for Tivantinib at 360 and Sorafenib at 400 mg BID

Sub-group	Tivantinib		Sorafenib	
	mean C_max_ (ng/mL)	AUC_0-12h_ (ng*hr/mL)	mean C_max_ (ng/mL)	AUC_0-12h_ (ng*hr/mL)
Non-HCC patients	1,323 ± 1,043	8,737 ± 10,759	6,757 ± 3,725	39,906 ± 20,604
HCC patients	1,510 ± 763	12,375 ± 7,496	5,920 ± 2,526	43,302 ± 20,863

In HCC patients receiving tivantinib at 360 mg BID and sorafenib at 400 mg BID, the mean C_max_ and AUC_0-12h_ of tivantinib in steady state (cycle 2) were 1,510 ± 763 ng/mL (*n* = 7) and 12,375 ± 7,496 ng*hr/mL (*n* = 6), respectively. For sorafenib, they were 5,920 ± 2,526 ng/mL (*n* = 7) and 43,302 ± 20,863 ng*hr/mL (*n* = 4), respectively.

Cycle 2 PK data are available for only one HCC patient who received the initial dose of tivantinib at 240 mg BID with sorafenib at 400 mg BID. This patient had a C_max_ of 879 ng/mL and an AUC0-12 h of 6,688 ng*hr/mL for tivantinib.

### MET status

The results of immunohistochemistry (IHC) tests for MET are summarized in Table 4. Twenty seven of 61 tested patients (44.3 %) were MET-high with the highest rate in RCC (73.7 %), followed by lung (50.0 %), HCC (40.0 %), and melanoma (28.6 %). All of the samples from breast cancer patients tested (8) were found to be MET-low.

**Table 4:** MET IHC Status*

Tumor type	No. patients tested	MET-High *N* (%)	MET-Low *N* (%)
All Patients	61	27 (44.3)	34 (55.7)
RCC	19	14 (73.7)	5 (26.3)
HCC	10	4 (40.0)	6 (60.0)
Melanoma	14	4 (28.6)	10 (71.4)
Breast	8	0 (0.0)	8 (100.0)
NSCLC	6	3 (50.0)	3 (50.0)
Others	4	2 (50.0)	2 (50.0)

*Sponsor’s IHC tests were done on small batches on an ongoing basis during the study. CLIA certified central laboratory IHC tests were performed as one batch at the end of patient enrollment. When discrepancy occurred, to avoid effects caused by MET degeneration over time, the sponsor′s results were used if the tissue sections tested by the central lab were prepared at the site and stored as slides

### Efficacy

The efficacy results are summarized in Table 5. The overall response rate (ORR) in all patients was 11.5 % with the highest ORR observed in melanoma (26.3 %), followed by RCC (15.0 %) and HCC (10.0 %). The disease control rate (DCR) (CR, PR and SD for at least 8 weeks) was 57.5 % in all patients, with the highest DCR seen in RCC (90.0 %), followed by HCC (65.0 %) and melanoma (63.2 %).

**Table 5:** RECIST Overall Objective Response, Disease Control Rate and by Patients’ MET Status

Total, *n* (%)	RCC (*N* = 20)	HCC (*N* = 20)	Melanoma (*N* = 19)	NSCLC (*N* = 12)	Breast (*N* = 10)	Other (*N* = 6)	All patients (*N* = 87)
CR	0 (0.0)	1 (5.0)	1 (5.3)*	0 (0.0)	0 (0.0)	0 (0.0)	2 (2.3)
PR	3 (15.0)	1 (5.0)	4 (21.1)	0 (0.0)	0 (0.0)	0 (0.0)	8 (9.2)
CR + PR median months on treatment (range)	26 (7, 27)	12 (9, 15)	6 (5, 13)				
SD for ≥ 8 weeks	15 (75.0)	11 (55.0)	7 (36.8)	1 (8.3)	4 (40.0)	2 (33.3)	40 (46.0)
SD for < 8 weeks	0 (0.0)	2 (10.0)	0 (0.0)	2 (16.7)	0 (0.0)	0	4 (4.6)
PD	2 (10.0)	4 (20.0)	5 (26.3)	8 (66.7)	4 (40.0)	2 (33.3)	25 (28.7)
Not evaluable	0 (0.0)	1 (5.0)	2 (10.5)	1 (8.3)	2 (20.0)	2 (33.3)	8 (9.2)
Overall response rate, %	3 (15.0)	2 (10.0)	5 (26.3)	0 (0.0)	0 (0.0)	0 (0.0)	10 (11.5)
Disease control rate (CR, PR & SD for ≥8 weeks) 8wkseast 8 weeks) at week 8, %	18 (90.0)	13 (65.0)	12 (63.2)	1 (8.3)	4 (40.0)	2 (33.3)	50 (57.5)
MET-high, *n*	(*N* = 14)	(*N* = 4)	(*N* = 4)	(*N* = 3)	(*N* = 0)	(*N* = 2)	(*N* = 27)
CR	0	1 (25.0)	0	0	0	0	1 (3.7)
PR	3 (21.4)	1 (25.0)	3 (75.0)	0	0	0	7 (25.9)
SD	11 (78.6)	1 (25.0)	1 (25.0)	0	0	0	13 (48.1)
PD	0	1 (25.0)	0	2 (66.7)	0	1 (50.0)	4 (14.8)
Not evaluable	0	0	0	1 (33.3)	0	1 (50.0)	2 (7.4)
Overall response rate, %	3 (21.4)	2 (50.0)	3 (75.0)	0	0	0	8 (29.6)
Disease control rate, %	14 (100)	3 (75.0)	4 (100.0)	0	0	0	21 (77.8)
MET-low, *n*	(*N* = 5)	(*N* = 6)	(*N* = 10)	(*N* = 3)	(*N* = 8)	(*N* = 2)	(*N* = 34)
CR	0	0	1 (10.0)	0	0	0	1 (2.9)
PR	0	0	1 (10.0)	0	0	0	1 (2.9)
SD	4 (80.0)	3 (50.0)	2 (20.0)	1 (33.3)	3 (37.5)	1 (50.0)	14 (41.2)
PD	1 (20.0)	2 (33.3)	4 (40.0)	2 (66.7)	3 (37.5)	1 (50.0)	13 (38.2)
Not evaluable	0	1 (16.7)	2 (20.0)	0	2 (25.0)	0	5 (14.7)
Overall response rate, %	0	0	2 (20.0)	0	0	0	2 (5.9)
Disease control rate, %	4 (80.0)	3 (50.0)	4 (40.0)	1 (33.3)	3 (37.5)	1 (50.0)	16 (47.1)
Prior anti-VEGF Tx, *n*	(*N* = 14)	(*N* = 8)					
CR	0	1 (12.5)					
PR	2 (14.3)	1 (12.5)					
SD	10 (71.4)	3 (37.5)					
PD	2 (14.3)	2 (25.0)					
Not evaluable	0	1 (12.5)					
Overall response rate, %	2 (14.3)	2 (25.0)					
Disease control rate, %	12 (85.7)	5 (62.5)					
No Prior anti-VEGF Tx, *n*	(*N* = 6)	(*N* = 12)					
CR	0	0					
PR	1 (16.7)	0					
SD	5 (83.3)	10 (83.3)					
PD	0	2 (16.7)					
Not evaluable	0	0					
Overall response rate, %	1 (16.7)	0					
Disease control rate, %	6 (100.0)	10 (83.3)					

Abbreviations: *CR* complete response; *PR* partial response; *SD* stable disease; *PD*: progressive disease

* The patient had one of the target lesions was a lymph node that regressed to <10 mm at the time of CR

In Table 5, the tumor response was further categorized by tumor type, MET status, and prior anti-VEGF treatment status. Despite the small number of patients enrolled per group, the DCR was higher in patients with MET-high compared to MET-low status in RCC, HCC, and melanoma; however, the differences were not statistically significant with *p*-values of 0.086 for RCC, 0.635 for HCC, and 0.083 for melanoma. In the RCC patients, the ORRs were similar (14.3 % vs. 16.7 %) between those who had (*n* = 14) or had not (*n* = 6) received prior anti-VEGF therapy. In the HCC patients, the ORRs were 25.0 % vs. 0 % between those who had (*n* = 8) or had not (*n* = 12) received prior anti-VEGF therapy. In melanoma patients, the ORRs were 20.0 % vs. 33.3 % between those with NRAS mutations (*n* = 10) and NRAS wild type/unknown (*n* = 9), respectively.

**Fig. 1:**
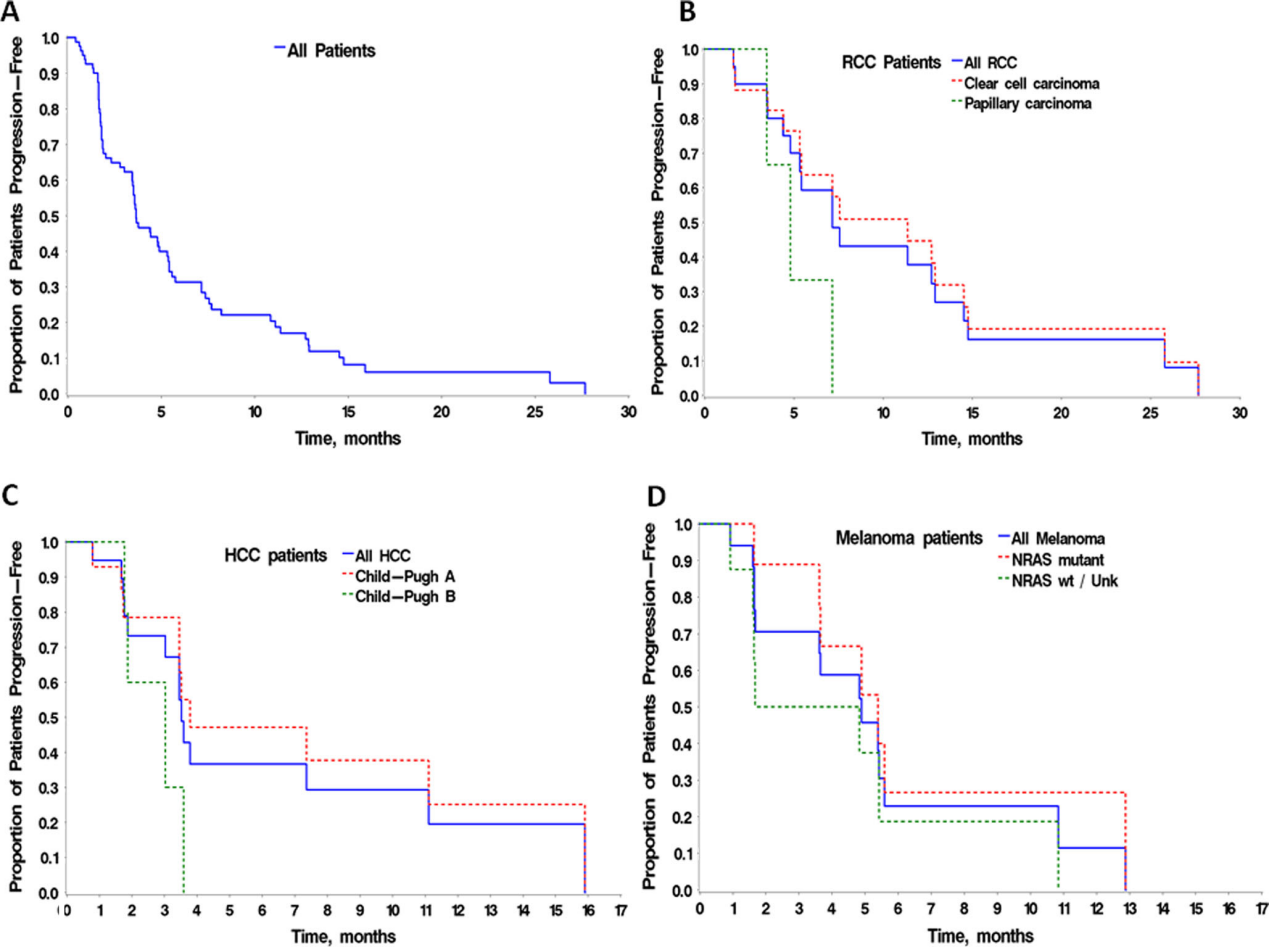
Kaplan-Meier plots showing (**a**) progression-free survival (PFS) in all patients (*n* = 87); (**b**) PFS in RCC patients (*n* = 20) by tumor subtype; (**c**) PFS in HCC patients (*n* = 20) by Child Pugh status; (**d**) PFS in melanoma patients (*n* = 19) by NRAS status

The median PFS (mPFS) was 3.6 months (95 % CI: 3.4–5.3 months) in all patients with the longest PFS observed in RCC patients (7.2 months, 95 % CI: 4.8–12.9 months), following by melanoma (4.9 months, 95 % CI: 1.7–5.6 months) and HCC (3.5 months, 95 % CI: 3.0–11.1 months) (Fig. 1). Seventeen of 20 RCC patients were clear cell subtype (including one with combination of clear cell and chromophobe histology) and three were papillary RCC. The patients with clear cell RCC had longer mPFS (11.4 months, 95 % CI: 5.3–14.5 months) compared to those with papillary RCC (4.8 months, 95 % CI: 3.5–7.2 months). In RCC patients, the mPFS was 7.6 months (95 % CI: 3.5–12.9 months) in those who had received prior anti-VEGF therapy (*n* = 14), which was similar to those who had not (7.2 months [95 % CI: 4.8–14.5], *n* = 6). In HCC patients, the PFS curve for patients with Child-Pugh A status appears to have a longer tail compared to that for patients with Child-Pugh B status with similar mPFS (3.8 [95 % CI:3.4–15.9] vs. 3.0 [95 % CI:1.8–3.6] months); HCC patients with at least one prior anti-VEGF therapy (*n* = 8) achieved longer mPFS compared to those without any prior anti-VEGF therapy (*n* = 12) (15.9 [95 % CI: 1.7–15.9] vs. 3.5 [95 % CI: 3.0–7.4] months), although small numbers prevent direct comparison.

For melanoma, the mPFS was 5.4 months in patients with NRAS mutation and 3.3 months in patients with NRAS wild type or NRAS status unknown.

## Discussion

In 2012, FDA approved cabozantinib, an inhibitor that targets VEGF, MET, and other tyrosine kinase pathways for the treatment of metastatic medullary thyroid cancer. Dual concomitant inhibition of MET and VEGF pathways remains an attractive anticancer strategy to further explore clinically [8, 10, 25, 26, 9]. Combining sorafenib and tivantinib, a VEGF inhibitor and a selective MET inhibitor, may provide synergistic or additive anti-tumor activity overcoming the resistance to sorafenib without causing possible off-target side effects of an unselective agent.

In this trial, treatment-related adverse events were predictable and primarily grade 1 and 2 with the most common (in ≥20 % patients) ones being rash, diarrhea, anorexia, fatigue, alopecia, palmar-plantar erythrodysaesthesia syndrome, and weight reduction. The RP2D for the combination therapy was tivantinib 360 mg BID plus sorafenib 400 mg BID for all tumor types except HCC. For HCC, the RP2D was tivantinib 240 mg BID plus sorafenib 400 mg BID. The increased rates of neutropenia reported in patients with HCC treated with tivantinib monotherapy [14, 13] and data from first 10 HCC patients in this study, suggested it would be prudent to start patients on a lower dose (240 mg BID) of tivantinib in HCC patients. The increased incidence of these side effects in HCC patients suggests higher tivantinib drug exposure likely due to the reduced drug clearance in patients compromised by hepatic cirrhosis.

According to our pharmacokinetic (PK) results, the tivantinib exposure in HCC patients was higher than that of other tumor types, but not as high as the exposure at same dose level in a phase 2 monotherapy study of tivantinib [14, 13]. The reason for this is unclear. Any conclusions should be made with caution as the PK data set for HCC patients in this study was small. In a couple of recent reports from in vitro studies, the cytotoxic activity of tivantinib independent of MET inhibition was observed via tubulin-related mechanisms of action [27, 28]. However, neurotoxicity, a hallmark of tubulin inhibitors, has not been reported either in this study or other clinical trials of tivantinib [15, 29, 11–14, 16, 30]. These indirect observations do not support a tubulin-mediated effect of tivantinib in the clinical setting.

MET-high rates for these patient groups were consistent with literature reports [31, 20, 32]. The high rate of MET-high status in RCC, HCC, and NSCLC confirmed in our study suggests a meaningful clinical framework worthy of further therapeutic investigation. In particular, a formal analysis of MET status in the setting of pre- and post-vascular endothelial growth factor receptor (VEGFR) treatment is warranted for elucidating the dynamic changes of MET status in the context of VEGFR resistance.

The ORR was highest in patients with melanoma (26 %), followed by RCC (15 %) and HCC (10 %). The DCR was highest in RCC (90 %), followed by HCC (65 %) and melanoma (63 %). Interestingly, all patients with RCC (*n* = 2) and HCC (*n* = 2) who achieved CR or PR were MET-high, as were three of five melanoma patients who had CR or PR. Among patients with MET-high status, 14 of 14 RCC, 4 of 4 melanoma, and 3 of 4 HCC patients achieved disease control. DCRs in MET-high patients were higher than those in MET-low patients with the same tumor type; however, the differences were not statistically significant. Despite the small number of tissue samples collected in this study, a correlation between baseline MET status and clinical response is suggested for the combination.

In the pivotal phase 3 study of sorafenib in RCC patients who received one prior systemic therapy, the mPFS was 167 days (5.5 months) in the sorafenib arm after progression on immunotherapy [33]. In the AXIS trial, mPFS after first line therapy containing sunitinib, bevacizumab plus interferon-alpha, temsirolimus, or cytokines was 6.7 months in axitinib arm and 4.7 months in sorafenib arm [29]. In our study, mPFS in 20 RCC patients was 7.2 months. This would be of clinical interest considering that 15 of 20 (75 %) of these patients had received at least one prior systemic therapy, including 14 (70 %) who had received at least one prior anti-VEGF therapy. The mPFS was 7.6 and 7.2 months in RCC patients with and without prior anti-VEGF therapy, respectively. Although the data from our phase 1 study cannot be directly compared with the phase 3 trial data, our results are encouraging.

Lastly, the HCC patients with Child-Pugh A status had a much longer PFS tail compared to that of patients with Child-Pugh B status. This may indicate that the combination treatment of tivantinib and sorafenib was not beneficial in Child-Pugh B HCC. This may hypothetically reflect the natural history of the disease, however, inferences are not conclusive due to the small number of patients in this group (*n* = 6).

In conclusion, in patients with advanced solid tumors, the combination therapy demonstrated a manageable and predictable safety profile and preliminary signs of anticancer activity, particularly in patients with RCC, HCC, and melanoma with MET-high status. The combination therapy was active in RCC and HCC patients regardless of prior exposure to VEGF inhibitor (s). In HCC patients, addition of tivantinib to sorafenib treatment led to disease stabilization and even clinical responses (CR and PR). These results provide additional impetus to further understand and develop treatment regimens that simultaneously attack the multiple compensation mechanisms that are acted upon targeted therapies.
